# Robust Switched Tracking Control for Wheeled Mobile Robots Considering the Actuators and Drivers

**DOI:** 10.3390/s18124316

**Published:** 2018-12-07

**Authors:** José Rafael García-Sánchez, Salvador Tavera-Mosqueda, Ramón Silva-Ortigoza, Victor Manuel Hernández-Guzmán, Jacobo Sandoval-Gutiérrez, Mariana Marcelino-Aranda, Hind Taud, Magdalena Marciano-Melchor

**Affiliations:** 1 Departamento de Procesos Productivos, Unidad Lerma, Universidad Autónoma Metropolitana, Estado de Mexico 52005, Mexico; j.sandoval@correo.ler.uam.mx; 2 Área de Mecatrónica & Energía Renovable, Centro de Innovación y Desarrollo Tecnológico en Cómputo, Instituto Politécnico Nacional, Ciudad de Mexico 07700, Mexico; staveram1500@alumno.ipn.mx (S.T.-M.); htaud@ipn.mx (H.T.); mmarciano@ipn.mx (M.M.-M.); 3 Facultad de Ingeniería, Universidad Autónoma de Querétaro, Querétaro 76010, Mexico; vmhg@uaq.mx; 4 Sección de Estudios de Posgrado e Investigación, Unidad Profesional Interdisciplinaria de Ingeniería y Ciencias Sociales y Administrativas, Instituto Politécnico Nacional, Ciudad de Mexico 08400, Mexico; mmarcelino@ipn.mx

**Keywords:** trajectory tracking, wheeled mobile robot, DC motor, DC/DC Buck power converter, hierarchical switched controller, hierarchical average controller, kinematic control, flat system, cascade switched control, sliding mode control, PI control

## Abstract

By using the hierarchical controller approach, a new solution for the control problem related to trajectory tracking in a differential drive wheeled mobile robot (DDWMR) is presented in this paper. For this aim, the dynamics of the three subsystems composing a DDWMR, i.e., the *mechanical structure* (differential drive type), the *actuators* (DC motors), and the *power stage* (DC/DC Buck power converters), are taken into account. The proposed hierarchical switched controller has three levels: the high level corresponds to a kinematic control for the mechanical structure; the medium level includes two controls based on differential flatness for the actuators; and the low level is linked to two cascade switched controls based on sliding modes and PI control for the power stage. The hierarchical switched controller was experimentally implemented on a DDWMR prototype via MATLAB-Simulink along with a DS1104 board. With the intention of assessing the performance of the switched controller, experimental results associated with a hierarchical average controller recently reported in literature are also presented here. The experimental results show the robustness of both controllers when parametric uncertainties are applied. However, the performance achieved with the switched controller introduced in the present paper is better than, or at least similar to, performance achieved with the average controller reported in literature.

## 1. Introduction

Over the past few years, the trajectory tracking problem in differential drive wheeled mobile robots (DDWMRs) has been one of the most challenging topics in robot control. This is mainly because DDWMRs are underactuated and non-holonomic systems [1,2,3]. This means that their theoretical nature is complex and, consequently, their control is not an easy task [4]. On the other hand, DDWMRs are composed, in general, of three subsystems: the *mechanical structure*, *actuators*, and *power stage*. In this direction, the design of tracking controls has been generally based on considering the kinematics/dynamics of the *mechanical structure*, and some other controls have also considered the dynamics of the *actuators*. On the other hand, the dynamics related to the *power stage* have been often neglected. However, when these three subsystems are considered in control design, according to [5,6,7], the performance of a DDWMR is better compared with the performance achieved with controllers that neglect the power stage. Moreover, if the dynamics of the power supply were also considered, an even better performance of the DDWMR could be achieved. Examples demonstrating this include the studies presented in [8,9,10,11], where the dynamics of the actuators, power stage, and electric power supply were taken into account in control design for mechatronic systems.

Regarding the design of control algorithms for the trajectory tracking task in DDWMRs, two approaches linked to the mathematical model of the mechanical structure are applied. These approaches are based on: (i) the kinematic model and (ii) the dynamic model. Thus, the literature review presented below is classified depending on the inclusion of the dynamics associated with the actuators and the power stage.

### 1.1. Control Algorithms Based on the Kinematic Model

This subsection presents the state of the art associated with the trajectory tracking task in DDWMRs when the kinematic model of the mechanical structure is used in control design. All papers related to such a topic can be classified into one of the following three categories: (i) Only kinematics of the mechanical structure, (ii) Kinematics of the mechanical structure + dynamics of the actuators, and (iii) Kinematics of the mechanical structure + dynamics of the actuators + dynamics of the power stage.

#### 1.1.1. Only Kinematics of the Mechanical Structure

Papers that consider only the kinematics of the mechanical structure in control design are the following. Mekhtiche et al. in [12] solved the tracking and obstacle avoidance problems by proposing a visual controller and a fuzzy controller, respectively. Li et al. proposed in [13] a unified tracking and regulation visual servoing strategy that uses Lyapunov-based techniques to compensate for unknown parameters. Simba et al. solved the trajectory generation problem and the obstacle avoidance problem via Bézier polynomials in [14]. Li et al. in [15] presented a visual servo strategy along with a pure rotation controller that solves the regulation problem. Chwa in [16] designed a sensorless robust backstepping-like feedback linearization control that compensates for the unknown velocities and kinematic disturbances. Li et al. in [17] proposed a teleoperation control law by using the time-domain passivity control approach that considers the slippage of the DDWMR. Chen et al. in [18] developed a tracking method that uses a B-spline algorithm to generate a smooth and obstacle-avoidance trajectory, while a genetic algorithm and a fuzzy control are combined for velocity control. Lages et al. in [19] proposed a non-smooth state feedback tracking control that generates a cloud of possible control signals, where the control to be applied is chosen depending on the regions in the control space. Xiao et al. in [20] designed a robust model predictive control scheme using neural network-based optimization to solve the trajectory tracking task. Škrjanc and Klančar in [21] designed a robust predictive control law, in a continuous space, whose design parameters are insensitive to the sampling period. Zhang et al. in [22] proposed a two-level robust tracking controller by using an adaptive control and the backstepping approach. Li et al. developed in [23] a teleoperation controller and an acceleration-level controller that allows a slave DDWMR to follow the master robot positions when slippage at the slave-level is considered. Seder et al. in [24] proposed a receding horizon control that generates feasible control sequences so that the DDWMR follows a desired trajectory, with lower computational costs. Lui et al. in [25] proposed a robust teleoperation control for two slave DDWMRs which cooperatively grasp and transport a deformable object while an operator, at the master site, receives visuo-haptic feedback. Zhao et al. in [26] designed a robust iterative learning control that exploits feedback-aided P-type learning terms to enhance the stability of the system when initial shift is considered. Rao et al. in [27] developed a control that combines fuzzy logic, neural network, and adaptive neuro-fuzzy inference system techniques with an integrated safe boundary algorithm to solve the tracking task. By considering the Takagi–Sugeno model, Sun et al. in [28] developed a fuzzy-model-based control where the position and posture of the DDWMR are estimated via visual odometry. Mu et al. in [29] designed a novel sliding mode control that is robust in the presence of matched and mismatched uncertainties. Liu et al. in [30] solved the tracking problem by developing a Lyapunov-based predictive tracking controller for DDWMRs subject to control input constraints. Sun et al. in [31] proposed a disturbance rejection model predictive control, with coupled input constraint and matched disturbances, along with two disturbance observers to estimate the unknown disturbances. Li et al. in [32] developed a visual servoing control to steer the DDWMR to a desired trajectory while an adaptive updating law, simultaneously, identifies the depth information. Alouache and Wu in [33] reported the design of a robust zero-order Takagi–Sugeno tracking controller that was deduced from a fuzzy proportional-derivative controller. Nascimento et al. in [34] presented a nonlinear model predictive control that uses a modified cost function with the aim of minimizing the difference between the DDWMR pose and orientation with respect to the reference trajectory’s pose and orientation, respectively. Li et al. in [35] designed a novel adaptive tracking control to estimate online from visual feedback of an omnidirectional camera, the position, orientation, and velocity of the DDWMR. Yang et al. in [36] proposed a robust model predictive control scheme composed of feedforward and feedback controllers which compensate for input increment constraints and external disturbances. Ke et al. in [37] developed a visual servoing control by using robust tube-based model predictive control for DDWMRs subject to bounded external disturbances.

#### 1.1.2. Kinematics of the Mechanical Structure + Dynamics of the Actuators

Tracking controls that take into account the kinematics of the mechanical structure and the dynamics of the actuators are presented here. C. Márquez-Sánchez et al. in [38] presented a two-level tracking controller composed of a kinematic control at the high level for the mechanical structure and a PI control at the low level for the actuators. Also, they reported a proposal for trajectory generation, where linear and angular velocities are defined via Bézier polynomials. Mu et al. in [39] developed a two-level tracking controller that is composed of a sliding mode control at the high level for the mechanical structure and a PI control at the low level for the actuators. Saleem et al. in [40] designed a controller of two levels; the high-level is an adaptive-fuzzy-tuned-proportional-derivative control law which receives visual feedback from an overhead camera, whereas two low-level PI controls are for the actuators.

#### 1.1.3. Kinematics of the Mechanical Structure + Dynamics of the Actuators + Dynamics of the Power Stage

Controls using the mathematical models of the three subsystems that compose a DDWMR are described in this section. Ortigoza et al. in [5] proposed, for the first time, a hierarchical controller of three levels which uses the dynamics of the mechanical structure, actuators, and power stage for solving the trajectory tracking task. Sánchez et al. reported in [6] the design of a three-level average tracking controller that considers the mechanical structure, actuators, and power stage. Also, an assessment of the controller performance, when its implementation is carried out via pulse width modulation (PWM) or sigma-delta modulation, was reported. García-Sánchez et al. in [7] developed a hierarchical tracking controller that takes into account the mathematical model of the mechanical structure, actuators, and power stage. The switched experimental implementation was carried out via sigma-delta modulation.

### 1.2. Control Algorithms Based on the Dynamic Model

This subsection presents a review of the literature associated with the tracking task in DDWMRs when the dynamic model of the mechanical structure is used in control design. Such a review is classified into the following two categories: (i) Only dynamics of the mechanical structure and (ii) Dynamics of the mechanical structure + dynamics of the actuators.

#### 1.2.1. Only Dynamics of the Mechanical Structure

Tracking controls that consider only the dynamics of the mechanical structure are the following. Roy and Kar in [41] proposed an adaptive robust outer-loop control that automatically tunes its gains in accordance with the tracking error, and it solves the tracking task when the DDWMR is subjected to time-varying input delay uncertainties. Huan et al. in [42] presented a robust control scheme that uses an adaptive virtual velocity control and a torque control along with a disturbance observer that estimates external disturbances and unknown parameters. Li et al. in [43] reported a hybrid intelligent algorithm based on a kinematic control and a fuzzy control that solves both the tracking and path-following control problems. Vos et al. in [44] presented a controller defined within the port-Hamiltonian framework; this was a combination of a heading control, a velocity tracking control, and a formation control for a group of DDWMRs. Rudra et al. in [45] designed a novel block-backstepping control that solves both the tracking and stabilization control problems in a DDWMR even when nonlinearities and coupling dynamics are considered in the mathematical model. Peng et al. in [46] investigated the formation control problem for multiple DDWMRs and designed kinematic controllers and neural network torque controllers for each robot. Lian et al. in [47] proposed a near-optimal tracking control based on a velocity-level backstepping kinematic controller and a receding horizon strategy that decompose the infinite-horizon optimal control problem into a series of finite-horizon optimal control problems. Sun et al. in [48] presented a robust tracking controller that not only improves the transient performance in the DDWMR but also significantly reduces the tracking errors by estimating the disturbances via two timescale filters. Chen in [49] designed a robust tracking control and a disturbance-observer-based virtual velocity control that considers the skidding, slipping, and input disturbance in the DDWMR. Peng and Shi in [50] reported a control strategy that integrates an auxiliary velocity controller along with an adaptive fuzzy integral terminal sliding mode controller for solving the tracking task, whereas the actuator voltage is used as the control input. Lashraki et al. in [51] developed a robust tracking controller (composed of a sliding mode control for tracking under disturbances) and robust saturation controllers (that handle the unmodeled dynamics and parameter uncertainties) for a two-docked mobile robotic system. Yue et al. in [52] developed a dynamic model that takes into account the slippage effects of the DDWMR. Also, they proposed a controller based on neural networks and a terminal sliding mode control for friction estimation and trajectory tracking of the robot. Capraro et al. in [53] proposed a control technique based on two-cascaded sliding mode controls which are combined with a feedback linearization controller and adaptive neural compensation, respectively. Chen et al. in [54] developed a nonlinear robust tracking control based on a feedback linearization controller and a robust compensator that also considers the unmodeled dynamics and modeling uncertainties. Nguyen and Le in [55] solved the tracking task via an adaptive tracking controller that is based on neural networks and considers wheel slippage, model uncertainties, and unknown bounded disturbances. Shen et al. in [56] designed an adaptive tracking control that considers the slipping/skidding dynamics associated with the DDWMR wheels and also the unknown mass center. Bian et al. in [57] reported a novel robust tracking control that combines the brain emotional learning (BEL) intelligent control method, which deals with the uncertainties and nonlinear terms of the system, along with the terminal sliding mode control method. Lastly, cases in which the dynamic model of the mechanical structure was used for control design were reported in [58,59].

#### 1.2.2. Dynamics of the Mechanical Structure + Dynamics of the Actuators

Papers reporting designs that use the dynamics of the mechanical structure and actuators are presented here. Hwang and Fang in [60] designed a robust fuzzy-adaptive hierarchical tracking control that uses the dynamics of the mechanical structure and the actuators. Kim and Kim in [61] proposed a time-optimal trajectory planning algorithm that includes the actuator dynamics, with an application intended for environments presenting multiple circular obstacles.

### 1.3. Discussion of Related Work, Motivation, and Contribution

The previous literature shows that, in general, the control design for the trajectory tracking task in DDWMRs has been tackled from five directions linked to the kinematics/dynamics of the mechanical structure: (1) by considering only the kinematics of the mechanical structure [12,13,14,15,16,17,18,19,20,21,22,23,24,25,26,27,28,29,30,31,32,33,34,35,36,37], (2) by taking into account the kinematics of the mechanical structure and the dynamics of the actuators [38,39,40], (3) by using the kinematic model of the mechanical structure along with the dynamics of the actuators and power stage [5,6,7], (4) by using only the dynamics of the mechanical structure [41,42,43,44,45,46,47,48,49,50,51,52,53,54,55,56,57,58,59], and (5) by considering the dynamics of the mechanical structure and the actuators [60,61]. Considering the aforementioned perspectives, the present paper is particularly motivated by (3), that is, when the mathematical models of the three subsystems composing a DDWMR are used in control design. In this direction, the main contribution of this paper is to present a new solution, compared with those reported in [5,6,7], for the tracking problem by designing a hierarchical switched controller that uses the mathematical models of the *mechanical structure*, *actuators*, and *power stage*. Such a controller has three levels: the control at *the high level* for the mechanical structure (differential drive type) is a kinematic control; at *the medium level* for the actuators (DC motors), two controls based on differential flatness are proposed; at *the low level* for the power stage (DC/DC Buck power converters), two cascade switched control schemes based on sliding mode control (SMC) and PI control were designed. Following the hierarchical controller approach, the controls at the high, medium, and low levels were interconnected to work as a whole. In order to enhance the contribution of this work, the performance of the switched controller was experimentally compared with results associated with the average controller reported in [7], which also considered the mathematical models of the *mechanical structure*, *actuators*, and *power stage* subsystems. In these experiments, parametric uncertainties were applied.

The remainder of this paper is organized as follows. The hierarchical switched controller design is described in Section 2, and its experimental implementation in a DDWMR prototype is presented in Section 3. Concluding remarks and a discussion of future research are given in Section 4.

## 2. Robust Hierarchical Switched Tracking Controller That Considers the Dynamics of All Subsystems Associated with a DDWMR

**Figure 1:**
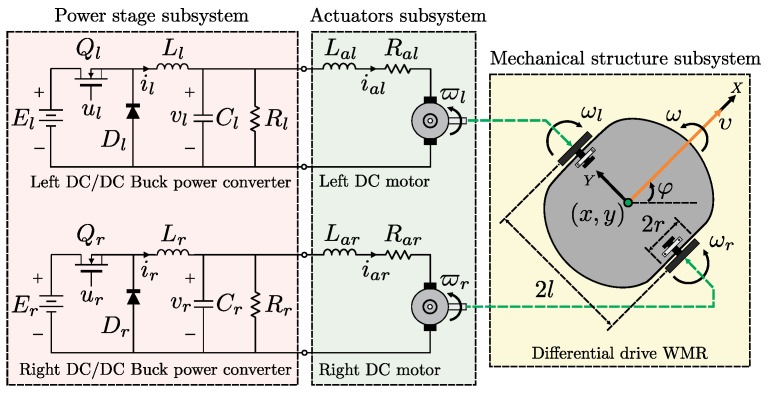
Differential drive wheeled mobile robot(DDWMR) diagram.

This section is devoted to the design of a hierarchical switched controller for the trajectory tracking task in a DDWMR, which is shown in Figure 1. It is worth mentioning that the parameters, components, and variables of the right wheel are indicated by the subscript *r* and those for the left wheel are indicated by the subscript *l*. The main characteristics of this hierarchical switched controller are as follows:

(1) In the high hierarchy level, a kinematic control, υ and ω, expressed in terms of $\omega_{r}$ and $\omega_{l}$ is proposed for the mechanical structure. This control allows the DDWMR to track a desired trajectory, i.e., $\left(x,y,\varphi \right)\to \left(x^{*},y^{*},\varphi^{*}\right)$, and also corresponds to the desired angular velocity profiles that the shafts of the DC motors have to track.
(2) In the medium hierarchy level, two controls based on differential flatness, $\vartheta_{r}$ and $\vartheta_{l}$, are designed so as to ensure that the shafts of the DC motors execute the angular velocity trajectory tracking task, i.e., $\left(\varpi_{r},\varpi_{l}\right)\to \left(\omega_{r},\omega_{l}\right)$. These controls also impose the desired voltage profiles that must be tracked by the output voltages of the DC/DC Buck power converters.
(3) In the low hierarchy level, via two cascade switched controls based on SMC and PI control, $v_{r}$ and $v_{l}$, it is assured that the output voltages of the DC/DC Buck power converters will track the desired voltage profiles imposed by the medium level, i.e., $\left(v_{r},v_{l}\right)\to \left(\vartheta_{r},\vartheta_{l}\right)$.
(4) By following the hierarchical controller approach, the controls described in previous items (1), (2), and (3) are interconnected so as to carry out the trajectory tracking task for the DDWMR.

### 2.1. High-Level Control

In this subsection, a kinematic control for a DDWMR is presented which allows for solving the trajectory tracking task, i.e., $\left(x,y,\varphi \right)\to \left(x^{*},y^{*},\varphi^{*}\right)$.

Assuming that the DDWMR moves in the XY plane and that the wheels move without slippage, then the kinematic model of the system is defined by [62]

(1) $$\begin{array}{cc} \dot{x} & =\upsilon \cos\varphi ,\\ \dot{y} & =\upsilon \sin\varphi ,\\ \dot{\varphi } & =\omega , \end{array}$$

with

(2) $$\begin{array}{c} \left[\begin{array}{c} \upsilon \\ \omega \end{array}\right]=\left[\begin{array}{cc} \frac{r}{2} & \frac{r}{2}\\ \frac{r}{2l} & -\frac{r}{2l} \end{array}\right]\left[\begin{array}{c} \omega_{r}\\ \omega_{l} \end{array}\right], \end{array}$$

where $\left(x,y\right)$ is the Cartesian position of the center of mass; φ is the orientation of the DDWMR; υ is the straight line velocity, ω is the angular velocity, and both are the inputs of the DDWMR; *r* is the ratio between the wheels, 2l is the distance between the wheels, and $\omega_{r}$, $\omega_{l}$ are the right and left angular velocities of the wheels, respectively (see Figure 1). In these equations, and in the remainder of this paper, the derivative with respect to time *t* will be denoted by a “dot” or by d/dt.

The objective of the kinematic control is to allow the DDWMR to achieve the desired trajectory imposed by a reference robot, i.e., $\left(x,y,\varphi \right)\to \left(x^{*},y^{*},\varphi^{*}\right)$. The kinematic model associated with the reference robot is given by

(3) $$\begin{array}{cc} {\dot{x}}^{*} & =\upsilon_{ref}\cos\varphi^{*},\\ {\dot{y}}^{*} & =\upsilon_{ref}\sin\varphi^{*},\\ {\dot{\varphi }}^{*} & =\omega_{ref}, \end{array}$$

where $x^{*}$, $y^{*}$, and $\varphi^{*}$ represent the configuration of the reference robot; $\upsilon_{ref}$ and $\omega_{ref}$ are its reference inputs. In accordance with [63], the following error signals are defined:

(4) $$\begin{array}{c} \left[\begin{array}{c} e_{1}\\ e_{2}\\ e_{3} \end{array}\right]=\left[\begin{array}{ccc} K_{2}\cos\varphi & K_{2}\sin\varphi & 0\\ -K_{2}\sin\varphi & K_{2}\cos\varphi & \alpha \\ 0 & 0 & 1 \end{array}\right]\left[\begin{array}{c} x^{*}-x\\ y^{*}-y\\ \varphi^{*}-\varphi \end{array}\right], \end{array}$$

with $K_{2}$ and α being positive constants. The time derivative of Equation (4) is determined by

(5) $$\begin{array}{cc} \frac{d}{dt}\left[\begin{array}{c} e_{1}\\ e_{2}\\ e_{3} \end{array}\right] & =\left[\begin{array}{cc} -K_{2} & e_{2}-\alpha e_{3}\\ 0-e_{1}-\alpha \\ 0 & -1 \end{array}\right]\left[\begin{array}{c} \upsilon \\ \omega \end{array}\right]+\left[\begin{array}{c} \upsilon_{ref}K_{2}\cos e_{3}\\ K_{2}\upsilon_{ref}\sin e_{3}+\alpha \omega_{ref}\\ \omega_{ref} \end{array}\right], \end{array}$$

where Equations (1) and (3) have been used. The following control inputs are taken from [64]

(6) $$\begin{array}{cc} \upsilon & =\upsilon_{ref}\cos e_{3}+K_{1}e_{1}, \end{array}$$

(7) $$\begin{array}{cc} \omega & =\omega_{ref}+\upsilon_{ref}K_{2}e_{2}+K_{3}\sin e_{3} \end{array}$$

with $K_{1}$ and $K_{3}$ being positive constants. In [63], it was shown that the controls in Equations (6) and (7) allow the error dynamics in Equation (5) to be asymptotically stable to the origin as long as $\alpha =1/K_{3}$ and $\upsilon^{*}>0$. This implies that the DDWMR in closed-loop with those control inputs achieves $\left(x,y,\varphi \right)\to \left(x^{*},y^{*},\varphi^{*}\right)$ when t→∞. Since it is assumed that the wheels of the robot are steered by DC motors, the control inputs in Equations (6) and (7) are transformed into the right and left angular velocities, respectively, as follows. Substituting Equations (6) and (7) into Equation (2), after some mathematical manipulation, the controls can now be expressed as

(8) $$\begin{array}{cc} \omega_{r} & =\frac{\upsilon_{ref}\left(\cos e_{3}+lK_{2}e_{2}\right)+l\left(\omega_{ref}+K_{3}\sin e_{3}\right)+K_{1}e_{1}}{r}, \end{array}$$

(9) $$\begin{array}{cc} \omega_{l} & =\frac{\upsilon_{ref}\left(\cos e_{3}-lK_{2}e_{2}\right)-l\left(\omega_{ref}+K_{3}\sin e_{3}\right)+K_{1}e_{1}}{r}. \end{array}$$

### 2.2. Medium-Level Control

In the previous subsection, the input signals $\omega_{r}$ and $\omega_{l}$, which are required by the DDWMR to achieve the trajectory tracking task, were found and are defined by Equations (8) and (9). Since DC motors are needed to steer the DDWMR, this subsection is focused on designing a control strategy so that the angular velocities of the DC motor shafts track the angular velocities imposed by the kinematic control of the DDWMR, i.e., $\left(\varpi_{r},\varpi_{l}\right)\to \left(\omega_{r},\omega_{l}\right)$.

The mathematical model of a DC motor expressed in terms of the angular velocity, ϖ, is defined by [62]

(10) $$\begin{array}{cc} L_{a}\frac{di_{a}}{dt} & =\vartheta -R_{a}i_{a}-k_{e}\varpi , \end{array}$$

(11) $$\begin{array}{cc} J\frac{d\varpi }{dt} & =-b\varpi +k_{m}i_{a}, \end{array}$$

with ϑ as the input voltage, $i_{a}$ as the armature current, $k_{e}$ as the counterelectromotive force constant, $k_{m}$ as the motor torque constant, $L_{a}$ as the armature inductance, $R_{a}$ as the armature resistance, *J* as the moment of inertia of the rotor and motor load, and *b* as the viscous friction coefficient of the rotor and motor load. As can be observed, parameters $k_{e}$, $k_{m}$, $L_{a}$, $R_{a}$, *J*, and *b* are required for control design purposes. In this direction, according to [7] and [65], the following first-order approximation of Equations (10) and (11), where the armature inductance $L_{a}$ has been neglected, is given by:

(12) $$\begin{array}{cc} \frac{d\varpi }{dt} & =-\zeta \varpi +\phi \vartheta , \end{array}$$

where

(13) $$\begin{array}{c} \zeta =\frac{1}{\tau },\;\phi =\frac{K}{\tau }, \end{array}$$

and

(14) $$\begin{array}{c} \tau =\frac{JR_{a}}{bR_{a}+k_{e}k_{m}},\;K=\frac{k_{m}}{bR_{a}+k_{e}k_{m}}. \end{array}$$

The parameters τ and *K* were experimentally obtained as in [7] and are given by,

(15) $$\begin{array}{c} \tau_{r}=20\times 10^{-3},\;K_{r}=634\times 10^{-3} \end{array}$$

for the right motor and

(16) $$\begin{array}{c} \tau_{l}=20\times 10^{-3},\;K_{l}=580\times 10^{-3} \end{array}$$

for the left motor. By using Equations (12), (13), (15), and (16), the mathematical models of the DC motors are determined by:

(17) $$\begin{array}{cc} \frac{d\varpi_{r}}{dt} & =-50\varpi_{r}+31.7\vartheta_{r},\;\frac{d\varpi_{l}}{dt}=-50\varpi_{l}+29\vartheta_{l}. \end{array}$$

On the other hand, with the aim of achieving $\left(\varpi_{r},\varpi_{l}\right)\to \left(\omega_{r},\omega_{l}\right)$, a differential flatness-based control [62] is now proposed for the model in Equation (12). In this direction, the dynamics in Equation (12) are rewritten in terms of the flat output ϖ as follows:

(18) $$\begin{array}{c} \vartheta =\frac{\dot{\varpi }+\zeta \varpi }{\phi }. \end{array}$$

A suitable definition for ϑ is

(19) $$\begin{array}{c} \vartheta =\frac{\delta +\zeta \varpi }{\phi }. \end{array}$$

After substituting Equation (19) into Equation (18), the tracking problem is reduced to controlling the following system:

(20) $$\begin{array}{c} \dot{\varpi }=\delta , \end{array}$$

where δ is an auxiliary control variable. In order to assure that $\varpi \to \varpi^{*}$ when t→∞, δ is proposed as:

(21) $$\begin{array}{c} \delta ={\dot{\varpi }}^{*}-k_{p}\left(\varpi -\varpi^{*}\right)-k_{i}\int_{0}^{t}\left(\varpi -\varpi^{*}\right)d\sigma , \end{array}$$

where $\varpi^{*}$ is the desired angular velocity and $\left(k_{p},k_{i}\right)$ are positive constants. When Equation (21) is placed into (20), the error tracking is defined as $e_{m}=\varpi -\varpi^{*}$, and the resultant expression is derived with respect to time; the following closed-loop error dynamics equation is obtained:

(22) $$\begin{array}{c} {\ddot{e}}_{m}+k_{p}{\dot{e}}_{m}+k_{i}e_{m}=0, \end{array}$$

whose characteristic polynomial is:

(23) $$\begin{array}{c} p_{c_{1}}\left(s\right)=s^{2}+k_{p}s+k_{i}. \end{array}$$

After Equation (23) is equated with the following stable polynomial:

(24) $$\begin{array}{c} p_{d_{1}}\left(s\right)=s^{2}+2\xi_{1}\omega_{n_{1}}s+\omega_{n_{1}}^{2}, \end{array}$$

where $\left(\xi_{1},\omega_{n_{1}}\right)>0$, it is found that gains $k_{p}$ and $k_{i}$ are given by:

(25) $$\begin{array}{c} k_{p}=2\xi_{1}\omega_{n_{1}},\;k_{i}=\omega_{n_{1}}^{2}. \end{array}$$

In this manner, the control in Equation (19) along with the gains in Equation (25) achieve $\varpi \to \varpi^{*}$ when t→∞.

### 2.3. Low-Level Control

The voltage inputs $\vartheta_{r}$ and $\vartheta_{l}$ required by the DC motors to achieve $\left(\varpi_{r},\varpi_{l}\right)\to \left(\omega_{r},\omega_{l}\right)$ were designed in the previous subsection and are given by Equation (19). Here, the design of a control algorithm that allows the output voltage of the Buck converters to track the desired voltage profiles imposed by the inputs of the DC motors, i.e., $\left(v_{r},v_{l}\right)\to \left(\vartheta_{r},\vartheta_{l}\right)$, is presented.

The schematic diagram of the DC/DC Buck power converter is shown in Figure 1. The switched dynamics of this converter is defined by [66]

(26) $$\begin{array}{cc} L\frac{di}{dt} & =-v+Eu, \end{array}$$

(27) $$\begin{array}{cc} C\frac{dv}{dt} & =i-\frac{v}{R}, \end{array}$$

with *i* being the current that flows through the inductor *L*, *v* is the converter output voltage at capacitor *C*, *R* is the load, *E* is the converter power supply, and *u* is a switched signal that takes values in the discrete set {0,1}. In order to ensure that the output voltage of the Buck converter tracks the desired voltage trajectory, i.e., $v\to v^{*}$, a cascade switched control based on an SMC and a PI control was proposed in [67] and is given by

(28) $$\begin{array}{cc} u & =\frac{1}{2}\left[1-\mathrm{sign}(s)\right] \end{array}$$

$$\begin{array}{c} s=i-i_{*},\;\mathrm{sign}\left(s\right)=\left\{\begin{array}{cc} +1, & \mathrm{si}\;s\geq 0,\\ -1, & \mathrm{si}\;s<0, \end{array}\right. \end{array}$$

(29) $$\begin{array}{c} i_{*}=C\frac{dv^{*}}{dt}+\frac{v^{*}}{R}+K_{p}e+K_{i}\int_{0}^{t}e\left(\tau \right)d\tau , \end{array}$$

(30) $$\begin{array}{c} e=v^{*}-v, \end{array}$$

where $i_{*}$ is the feedback reference current, *E*, *v*, $v^{*}$, *i*, and *u* were defined previously, and *e* is the voltage error. As was proved in [67], the system described in Equations (26) and (27) in closed-loop with Equation (28) is asymptotically stable as long as $K_{p}$ and $K_{i}$ are positive.

### 2.4. Hierarchical Switched Tracking Controller Design

By following the hierarchical controller approach (see [5,6,7]), in this subsection, the controls presented in Section 2.1, Section 2.2 and Section 2.3 are interconnected so that the trajectory tracking task in DDWMR can be solved.

Considering the kinematic model of the DDWMR, Equation (1), it was found that control inputs $\omega_{r}$ and $\omega_{l}$, defined by Equations (8) and (9), allow the DDWMR to track the reference mobile robot defined in Equation (3). Two DC motors were used to steer the DDWMR. Thus, from Equations (19) and (21), the control inputs $\vartheta_{r}$ and $\vartheta_{l}$ ensuring that $\left(\varpi_{r},\varpi_{l}\right)\to \left(\omega_{r},\omega_{l}\right)$ are given by

(31) $$\begin{array}{c} \vartheta_{r}=\frac{\delta_{r}+\zeta_{r}\varpi_{r}}{\phi_{r}},\;\delta_{r}={\dot{\varpi }}_{r}^{*}-k_{p_{r}}\left(\varpi_{r}-\varpi_{r}^{*}\right)-k_{i_{r}}\int_{0}^{t}\left(\varpi_{r}-\varpi_{r}^{*}\right)d\sigma_{r}, \end{array}$$

and

(32) $$\begin{array}{c} \vartheta_{l}=\frac{\delta_{l}+\zeta_{l}\varpi_{l}}{\phi_{l}},\;\delta_{l}={\dot{\varpi }}_{l}^{*}-k_{p_{l}}\left(\varpi_{l}-\varpi_{l}^{*}\right)-k_{i_{l}}\int_{0}^{t}\left(\varpi_{l}-\varpi_{l}^{*}\right)d\sigma_{l}, \end{array}$$

for the right and left motors, respectively. In such controls,

(33) $$\begin{array}{cc} \left(\varpi_{r}^{*},\varpi_{l}^{*}\right) & =\left(\omega_{r},\omega_{l}\right). \end{array}$$

Taking into account that each DC motor is driven by a Buck power converter, from Equation (28), it was found that the switched controls $u_{r}$ and $u_{l}$ that accomplish $\left(v_{r},v_{l}\right)\to \left(\vartheta_{r},\vartheta_{l}\right)$ are expressed as

(34) $$\begin{array}{c} u_{r}=\frac{1}{2}\left[1-\mathrm{sign}\left(s_{r}\right)\right],\;s_{r}=i_{r}-i_{{*}_{r}}, \end{array}$$

(35) $$\begin{array}{c} u_{l}=\frac{1}{2}\left[1-\mathrm{sign}\left(s_{l}\right)\right],\;s_{l}=i_{l}-i_{{*}_{l}}, \end{array}$$

for the right and left Buck power converters, respectively. In these controls,

(36) $$\begin{array}{cc} \left(v_{r}^{*},v_{l}^{*}\right) & =\left(\vartheta_{r},\vartheta_{l}\right). \end{array}$$

**Figure 2:**
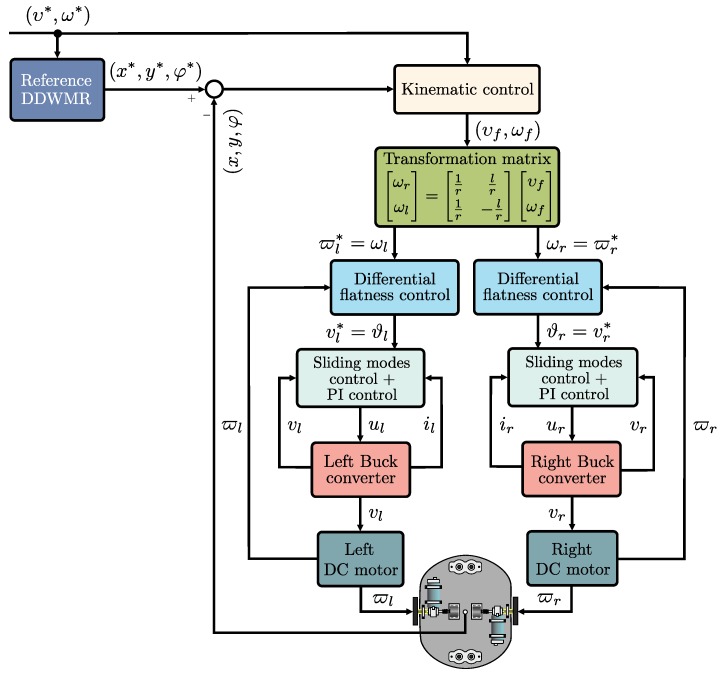
Block diagram of the hierarchical switched controller in closed-loop with the DDWMR.

In brief, the switched controls, Equations (34) and (35), allow for $\left(v_{r},v_{l}\right)\to \left(\vartheta_{r},\vartheta_{l}\right)$, thus $\left(\varpi_{r},\varpi_{l}\right)\to \left(\omega_{r},\omega_{l}\right)$. In consequence, the control objective of the DDWMR is achieved since $\left(x,y,\varphi \right)\to \left(x^{*},y^{*},\varphi^{*}\right)$. Figure 2 depicts the integration of the hierarchical switched controller in closed-loop with the DDWMR.

## 3. Experimental Results

In order to enhance the contribution of this paper, the hierarchical switched controller developed in Section 2 was experimentally compared with the hierarchical average controller recently reported in [7]. Thus, this section presents the experimental implementation of the aforementioned controllers.

### 3.1. Controllers to be Experimentally Implemented

The generalities of the controllers to be experimentally assessed are presented in this subsection. Firstly, the hierarchical switched controller developed in this study is described and, secondly, the hierarchical average controller reported in [7] is shown.

- Hierarchical switched controller (developed in Section 2). This controller is composed by the following three stages.
  *High level*: Mechanical structure
  (37) $$\begin{array}{c} \omega_{r}=\frac{\upsilon_{ref}\left(\cos e_{3}+lK_{2}e_{2}\right)+l\left(\omega_{ref}+K_{3}\sin e_{3}\right)+K_{1}e_{1}}{r}, \end{array}$$
  (38) $$\begin{array}{c} \omega_{l}=\frac{\upsilon_{ref}\left(\cos e_{3}-lK_{2}e_{2}\right)-l\left(\omega_{ref}+K_{3}\sin e_{3}\right)+K_{1}e_{1}}{r}, \end{array}$$
  where $\left(K_{1},K_{2},K_{3}\right)>0$.
  *Medium level*: Actuators
  (39) $$\begin{array}{c} \vartheta_{r}=\frac{\delta_{r}+\zeta_{r}\varpi_{r}}{\phi_{r}},\;\delta_{r}={\dot{\varpi }}_{r}^{*}-k_{p_{r}}\left(\varpi_{r}-\varpi_{r}^{*}\right)-k_{i_{r}}\int_{0}^{t}\left(\varpi_{r}-\varpi_{r}^{*}\right)d\sigma_{r}, \end{array}$$
  (40) $$\begin{array}{c} \vartheta_{l}=\frac{\delta_{l}+\zeta_{l}\varpi_{l}}{\phi_{l}},\;\delta_{l}={\dot{\varpi }}_{l}^{*}-k_{p_{l}}\left(\varpi_{l}-\varpi_{l}^{*}\right)-k_{i_{l}}\int_{0}^{t}\left(\varpi_{l}-\varpi_{l}^{*}\right)d\sigma_{l}, \end{array}$$
  and the gains $k_{p_{r}},k_{i_{r}},k_{p_{l}},k_{i_{l}}$ were found to be
  $$\begin{array}{c} k_{p_{r}}=2\xi_{1_{r}}\omega_{n_{1_{r}}},\;k_{i_{r}}=\omega_{n_{1_{r}}}^{2},\;k_{p_{l}}=2\xi_{1_{l}}\omega_{n_{1_{l}}},\;k_{i_{l}}=\omega_{n_{1_{l}}}^{2}. \end{array}$$
  *Low level*: Power stage
  (41) $$\begin{array}{c} u_{r}=\frac{1}{2}\left[1-\mathrm{sign}\left(s_{r}\right)\right],\;s_{r}=i_{r}-i_{{*}_{r}}, \end{array}$$
  (42) $$\begin{array}{c} u_{l}=\frac{1}{2}\left[1-\mathrm{sign}\left(s_{l}\right)\right],\;s_{l}=i_{l}-i_{{*}_{l}}, \end{array}$$
  where
  $$\begin{array}{c} i_{{*}_{r}}=C_{r}\frac{dv_{r}^{*}}{dt}+\frac{v_{r}^{*}}{R_{r}}+K_{p_{r}}e_{r}+K_{i_{r}}\int_{0}^{t}e_{r}\left(\tau \right)d\tau_{r},\;e_{r}=v_{r}^{*}-v_{r},\\ i_{{*}_{l}}=C_{l}\frac{dv_{l}^{*}}{dt}+\frac{v_{l}^{*}}{R_{l}}+K_{p_{l}}e_{l}+K_{i_{l}}\int_{0}^{t}e_{l}\left(\tau \right)d\tau_{l},\;e_{l}=v_{l}^{*}-v_{l}, \end{array}$$
  and the gains $K_{p_{r}},K_{i_{r}},K_{p_{l}},K_{i_{l}}$ are positive.
- Hierarchical average controller (reported in [7]). This controller also comprises three levels of control: *high level* for the mechanical structure, *medium level* for the actuators, and *low level* for the power stage. The controls associated with the *high level* and the *medium level* correspond to Equations (37)–(40), respectively. On the other hand, the control related to the *low level* is given by
  (43) $$u_{av_{r}}=\frac{L_{r}C_{r}}{E_{r}}\eta_{r}+\frac{L_{r}}{R_{r}E_{r}}{\dot{v}}_{r}+\frac{1}{E_{r}}v_{r},\;\eta_{r}={\ddot{v}}_{r}^{*}-\beta_{2_{r}}\;\left({\dot{v}}_{r}-{\dot{v}}_{r}^{*}\right)-\beta_{1_{r}}\;\left(v_{r}-v_{r}^{*}\right)-\beta_{0_{r}}\;\int_{0}^{t}\;\left(v_{r}-v_{r}^{*}\right)\;d\sigma_{r},$$
  (44) $$u_{av_{l}}=\frac{L_{l}C_{l}}{E_{l}}\eta_{l}+\frac{L_{l}}{R_{l}E_{l}}{\dot{v}}_{l}+\frac{1}{E_{l}}v_{l},\;\eta_{l}={\ddot{v}}_{l}^{*}-\beta_{2_{l}}\left({\dot{v}}_{l}-{\dot{v}}_{l}^{*}\right)-\beta_{1_{l}}\left(v_{l}-v_{l}^{*}\right)-\beta_{0_{l}}\int_{0}^{t}\left(v_{l}-v_{l}^{*}\right)d\sigma_{l},$$
  and the gains $\beta_{2_{r}},\beta_{1_{r}},\beta_{0_{r}},\beta_{2_{l}},\beta_{1_{l}}$, and $\beta_{0_{l}}$ are defined as
  $$\begin{array}{c} \beta_{2_{r}}=a_{2_{r}}+2\xi_{2_{r}}\omega_{n_{2_{r}}},\;\beta_{1_{r}}=2\xi_{2_{r}}\omega_{n_{2_{r}}}a_{2_{r}}+\omega_{n_{2_{r}}}^{2},\;\beta_{0_{r}}=a_{2_{r}}\omega_{n_{2_{r}}}^{2},\\ \beta_{2_{l}}=a_{2_{l}}+2\xi_{2_{l}}\omega_{n_{2_{l}}},\;\beta_{1_{l}}=2\xi_{2_{l}}\omega_{n_{2_{l}}}a_{2_{l}}+\omega_{n_{2_{l}}}^{2},\;\beta_{0_{l}}=a_{2_{l}}\omega_{n_{2_{l}}}^{2}. \end{array}$$

### 3.2. Experimental Prototype

**Figure 3:**
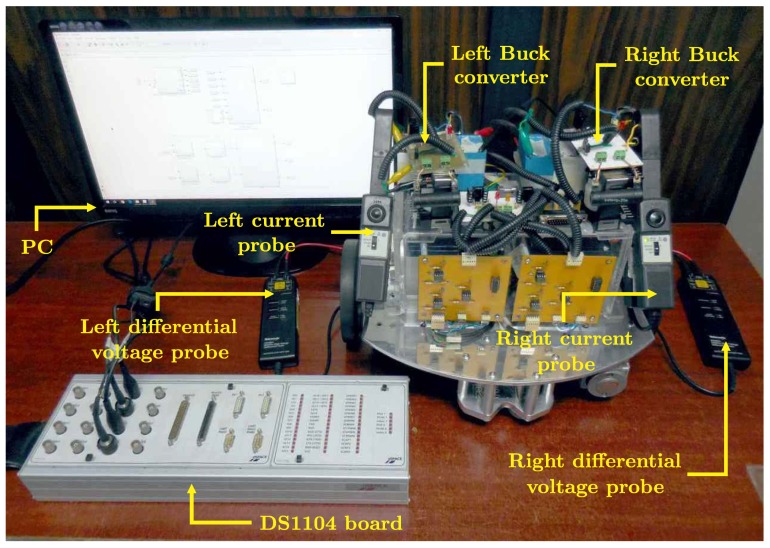
DDWMR prototype.

The experimental implementation of the hierarchical switched controller and the hierarchical average controller was carried out via MATLAB-Simulink, the real-time interface ControlDesk, and the DS1104 board on the DDWMR prototype shown in Figure 3. The prototype is $422\times 10^{-3}\;m$ in length, $350\times 10^{-3}\;m$ in width, $350\times 10^{-3}\;m$ in height, and its mass is 30kg. Each wheel is steered by a DC motor GNM3150 + G2.6 (provided with a 30:1 gearbox) which is driven by a DC/DC Buck power converter.

**Figure 4:**
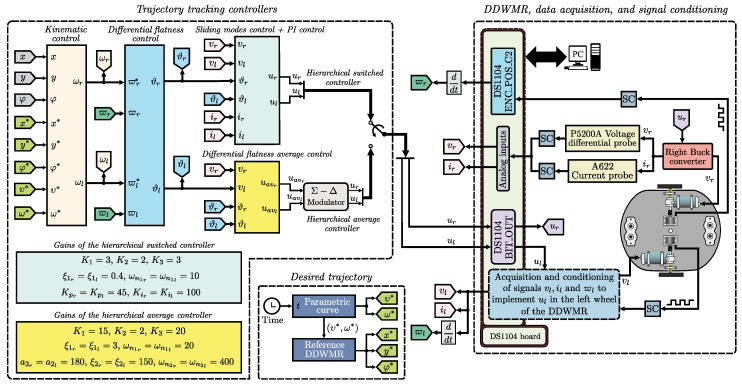
Connections diagram of the DDWMR in closed-loop.

The connections diagram of the DDWMR in closed-loop, when either the hierarchical switched controller or the hierarchical average controller is selected, with MATLAB-Simulink and the DS1104 board is depicted in Figure 4.

The diagram of Figure 4 consists of three blocks, which are described below.

- Trajectory tracking controllers. The synthesis and programming of the hierarchical switched controller, Equations (37)–(42), and the hierarchical average controller, Equations (37)–(40), (43), and (44), were carried out as described here via MATLAB-Simulink. In this block, the following six sub-blocks can be observed:
  *(1) Kinematic control*. This control corresponds to the high level of both hierarchical controllers. It is given by Equations (37) and (38) and requires the following information associated with the DDWMR:
  r=0.075m,l=0.19m.
  *(2) Differential flatness control*. This is related to the medium-level control of both hierarchical controllers. It is defined by Equations (39) and (40) and requires the parameters given by (15) and (16). That is,
  $$\tau_{r}=20\times 10^{-3},\;K_{r}=634\times 10^{-3},\;\tau_{l}=20\times 10^{-3},\;K_{l}=580\times 10^{-3}.$$
  *(3) Sliding mode control + PI control*. This control is associated with the low level of the hierarchical switched controller, Equations (41) and (42), and considers some parameters of the DC/DC Buck power converters. Such parameters are
  $$C_{r}=C_{l}=220\times 10^{-6}\;F,\;R_{r}=R_{l}=100\;\Omega .$$
  *(4) Differential flatness average control*. Corresponds to the low level of the hierarchical average controller reported in [7], Equations (43) and (44), and uses all parameters of the DC/DC Buck power converters. Those parameters are
  $$\begin{array}{c} C_{r}=C_{l}=220\times 10^{-6}\;F,\;R_{r}=R_{l}=100\;\Omega ,\\ L_{r}=10.129\times 10^{-3}\;H,\;L_{l}=10.6\times 10^{-3}\;H,\;E_{r}=E_{l}=28\;V. \end{array}$$
  It is worth noting that the hierarchical average controller, composed of the previous items (1), (2), and (4), was designed on the basis of the average model associated with the power stage (DC/DC Buck power converters). Because of this, a modulator is required for its appropriate experimental implementation. In this direction, the sigma-delta modulator (Σ−Δ-modulator) was used in order to make a fair comparison between both controllers, i.e., the switched one and the average one.
  *(5) Gains of the hierarchical switched controller*. Here, the gains associated with the controls of the high, medium, and low levels are specified. For the high level, the gains were chosen as
  $$K_{1}=3,\;K_{2}=2,\;K_{3}=3.$$
  Meanwhile, the gains linked to the medium level, i.e., $\left(k_{p_{r}},k_{i_{r}},k_{p_{l}},k_{i_{l}}\right)$, were obtained by choosing their parameters as follows:
  $$\xi_{1_{r}}=\xi_{1_{l}}=0.4,\;\omega_{n_{1_{r}}}=\omega_{n_{1_{l}}}=10.$$
  Lastly, the gains of the low level were proposed as
  $$K_{p_{r}}=K_{p_{l}}=45,\;K_{i_{r}}=K_{i_{l}}=100.$$
  *(6) Gains of the hierarchical average controller*. In this block, the gains of the three levels of control (high, medium, and low) are defined. For the high level, the gains were selected as
  $$K_{1}=15,\;K_{2}=2,\;K_{3}=20.$$
  On the other hand, the gains $\left(k_{p_{r}},k_{i_{r}},k_{p_{l}},k_{i_{l}}\right)$, related to the medium level, were found by choosing the following parameters:
  $$\xi_{1_{r}}=\xi_{1_{l}}=3,\;\omega_{n_{1_{r}}}=\omega_{n_{1_{l}}}=20.$$
  Lastly, the gains of the low level, i.e., $\left(\beta_{2_{r}},\beta_{1_{r}},\beta_{0_{r}},\beta_{2_{l}},\beta_{1_{l}},\beta_{0_{l}}\right)$, were determined by selecting their parameters,
  $$a_{2_{r}}=a_{2_{l}}=180,\;\xi_{2_{r}}=\xi_{2_{l}}=150,\;\omega_{n_{2_{r}}}=\omega_{n_{2_{l}}}=400.$$
- Desired trajectory. The results presented in this paper are applied using the following Bézier polynomials to obtain the reference velocities $\upsilon_{ref}$ and $\omega_{ref}$:
  (45) $$\begin{array}{c} p_{1}\left(t\right)={\bar{\upsilon }}_{ref}\left(t_{i}\right)+\;\left[{\bar{\upsilon }}_{ref}\left(t_{f}\right)-{\bar{\upsilon }}_{ref}\left(t_{i}\right)\right]\psi \left(t,t_{i},t_{f}\right), \end{array}$$
  (46) $$\begin{array}{c} p_{2}\left(t\right)={\bar{\omega }}_{ref}\left(t_{i}\right)+\;\left[{\bar{\omega }}_{ref}\left(t_{f}\right)-{\bar{\omega }}_{ref}\left(t_{i}\right)\right]\psi \left(t,t_{i},t_{f}\right), \end{array}$$
  where $t_{i}$ and $t_{f}$ are the initial and final times of the given trajectory, the pairs $\left[{\bar{\upsilon }}_{ref}\left(t_{i}\right),{\bar{\upsilon }}_{ref}\left(t_{f}\right)\right]$ and $\left[{\bar{\omega }}_{ref}\left(t_{i}\right),{\bar{\omega }}_{ref}\left(t_{f}\right)\right]$ represent the transference linear and angular velocities related to $t_{i}$ and $t_{f}$, and $\psi (t,t_{i},t_{f})$ is a polynomial function given by
  (47) $$\begin{array}{cc} \psi (t,t_{i},t_{f}) & ={\left(\frac{t-t_{i}}{t_{f}-t_{i}}\right)}^{3}\times \left[10-15\left(\frac{t-t_{i}}{t_{f}-t_{i}}\right)+6{\left(\frac{t-t_{i}}{t_{f}-t_{i}}\right)}^{2}\right]. \end{array}$$
  Through Equations (45) and (46), the reference velocities $\upsilon_{ref}$ and $\omega_{ref}$ were generated according to Table 1. Thus, by using Equation (3), the trajectory to be tracked by the DDWMR in the XY plane, i.e., $\left(x^{*},y^{*},\varphi^{*}\right)$, is found. On the other hand, after substituting $\upsilon_{ref}$ and $\omega_{ref}$ in (2), and after some algebraic manipulation, $\omega_{r_{ref}}$ and $\omega_{l_{ref}}$ are found.
- DDWMR, data acquisition, and signal conditioning. This block shows the connections between the DS1104 board and the DDWMR. The voltages $\left(v_{r},v_{l}\right)$, currents $\left(i_{r},i_{l}\right)$, and angular velocities $\left(\varpi_{r},\varpi_{l}\right)$ are acquired via two Tektronix P5200A voltage probes, two Tektronix A622 current probes, and two Omron E6B2-CWZ6C incremental encoders, respectively. As can be observed, signal conditioning (SC) is performed in each signal.

### 3.3. Experimental Results Related to the Controllers

In this subsection, the experimental results of the switched controller introduced in the present paper, Equations (37)–(42), and those associated with the hierarchical average controller reported in [7], Equations (37)–(40), (43), and (44), are presented.

The experimental results show a visual assessment of both controllers when they are implemented on the DDWMR depicted in Figure 3. In such experiments, the results related to the hierarchical switched controller correspond to $y_{s}\left(x_{s}\right),\varphi_{s},\varpi_{r_{s}},\varpi_{l_{s}},v_{r_{s}},v_{l_{s}},i_{r_{s}},i_{l_{s}},u_{r_{s}}$, and $u_{l_{s}}$. On the other hand, in the same experiments, the results of the hierarchical average controller correspond to $y_{av}\left(x_{av}\right),\varphi_{av},\varpi_{r_{av}},\varpi_{l_{av}},v_{r_{av}},v_{l_{av}},i_{r_{av}},i_{l_{av}},u_{r_{av}}$, and $u_{l_{av}}$. Note that the experimental implementation of the DDWMR in closed-loop (with both controllers) considers parametric uncertainties in loads $\left(R_{r},R_{l}\right)$ and power supplies $\left(E_{r},E_{l}\right)$ associated with the DC/DC Buck power converters. Such variations should be taken into account in control design, since they are the most common changes in power converters.

#### 3.3.1. Experiment 1: Results Associated with Abrupt Changes in Loads

**Figure 5:**
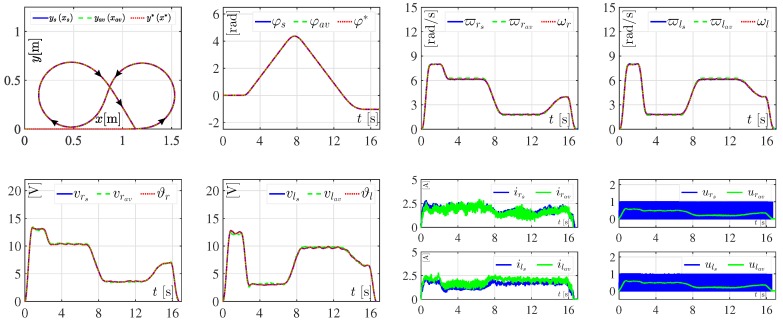
Experimental results in closed-loop when abrupt changes in $R_{r}$ and $R_{l}$ appear. The results associated with the hierarchical switched controller are denoted as $y_{s}\left(x_{s}\right)$, $\varphi_{s}$, $\varpi_{r_{s}}$, $\varpi_{l_{s}}$, $v_{r_{s}}$, $v_{l_{s}}$, $i_{r_{s}}$, $i_{l_{s}}$, $u_{r_{s}}$, and $u_{l_{s}}$. The results related to the hierarchical average controller are labeled as $y_{av}\left(x_{av}\right)$, $\varphi_{av}$, $\varpi_{r_{av}}$, $\varpi_{l_{av}}$, $v_{r_{av}}$, $v_{l_{av}}$, $i_{r_{av}}$, $i_{l_{av}}$, $u_{r_{av}}$, and $u_{l_{av}}$.

The performance of the DDWMR in closed-loop with both the hierarchical switched controller and the hierarchical average controller was assessed by introducing the abrupt variations listed in Table 2 to $R_{r}$ and $R_{l}$. The corresponding results of this experiment are shown in Figure 5.

As can be observed in Figure 5, the control objective is successfully accomplished using either the hierarchical switched controller or the hierarchical average controller. Both controllers, in general, are robust since they achieve $\left(x,y,\varphi \right)\to \left(x^{*},y^{*},\varphi^{*}\right)$, even when the abrupt variations from Table 2 are taken into account. However, small tracking errors related to the average angular velocities (i.e., $\varpi_{r_{av}}$ and $\varpi_{l_{av}}$) and the average voltages (i.e., $v_{r_{av}}$ and $v_{l_{av}}$) can be observed. Apparently, such small tracking errors are because the low level control (i.e., the differential flatness control) of the hierarchical average controller is less robust compared with the low level control (i.e., the SMC plus PI control) of the hierarchical switched controller. On the other hand, in Figure 5, it is also observed that controls $u_{r_{av}}$ and $u_{l_{av}}$ are never saturated.

#### 3.3.2. Experiment 2: Results Associated with Abrupt Changes in Power Supplies

**Figure 6:**
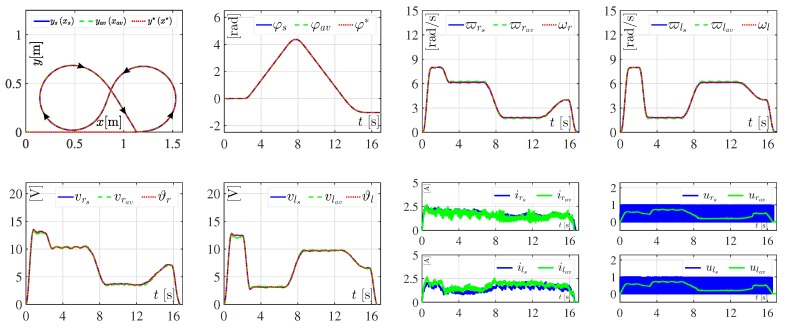
Experimental results in closed-loop when variations in $E_{r}$ and $E_{l}$ appear. The corresponding results of the hierarchical switched controller are represented by $y_{s}\left(x_{s}\right)$, $\varphi_{s}$, $\varpi_{r_{s}}$, $\varpi_{l_{s}}$, $v_{r_{s}}$, $v_{l_{s}}$, $i_{r_{s}}$, $i_{l_{s}}$, $u_{r_{s}}$, and $u_{l_{s}}$. The results associated with the hierarchical average controller are denoted by $y_{av}\left(x_{av}\right)$, $\varphi_{av}$, $\varpi_{r_{av}}$, $\varpi_{l_{av}}$, $v_{r_{av}}$, $v_{l_{av}}$, $i_{r_{av}}$, $i_{l_{av}}$, $u_{r_{av}}$, and $u_{l_{av}}$.

With the aim of assessing the performance in closed-loop of both the hierarchical switched controller and the hierarchical average controller, the abrupt variations from Table 3 were introduced to $E_{r}$ and $E_{l}$. The results associated with this experiment are depicted in Figure 6.

Figure 6 shows, again, that the control objective is successfully achieved with either the hierarchical switched controller or the hierarchical average controller. That is, $\left(x,y,\varphi \right)\to \left(x^{*},y^{*},\varphi^{*}\right)$ when the abrupt variations from Table 3 are considered. The small tracking errors related to the average angular velocities (i.e., $\varpi_{r_{av}}$ and $\varpi_{l_{av}}$) and the average voltages (i.e., $v_{r_{av}}$ and $v_{l_{av}}$) are due to the aforementioned issues from the section on Experiment 1. Also, it is observed that controls $u_{r_{av}}$ and $u_{l_{av}}$ are never saturated, as in the previous experiment.

According to the experimental results, when abrupt changes are introduced into the system, it is observed that the hierarchical switched controller exhibits a better performance than, or at least similar to, the performance achieved with the hierarchical average controller. Likewise, the experimental implementation of the latter requires the knowledge of all parameters associated with the power converters.

## 4. Conclusions

The design of a new robust hierarchical switched controller for the trajectory tracking task in a DDWMR is introduced in this paper. Such a controller considers the dynamics associated with the three subsystems that compose a DDWMR, i.e., the mechanical structure, the actuators, and the power stage. Three levels were proposed for the hierarchical switched controller: the high level is a kinematic control for the mechanical structure, the medium level are two controls based on differential flatness for the actuators, and the low level are two cascade switched controls based on SMC plus PI control for the power stage.

The hierarchical switched controller was experimentally assessed with the hierarchical average controller recently reported in [7]. Both controllers were tested on a DDWMR prototype through MATLAB-Simulink, the real-time interface ControlDesk, and a DS1104 board from dSPACE. According to the experimental results, the controllers not only solved the control objective, i.e., $\left(x,y,\varphi \right)\to \left(x^{*},y^{*},\varphi^{*}\right)$, but also their robustness was shown by introducing abrupt and simultaneous changes in some parameters. However, the performance achieved with the switched controller introduced in the present paper is better than, or at least similar to, performance achieved with the average controller presented in [7]. In this direction, it is worth emphasizing that, unlike the controller designed in [7], the one proposed in this paper is more suitable to be experimentally implemented on the DDWMR. This is because the subsystem power stage is a variable structure system and a switched control fits better than an average control, since no type of modulation is required compared with the latter.

Motivated by the obtained results, future research will be devoted to solving other important tasks in mobile robotics, such as obstacle avoidance and path tracking. In other directions, another future study could focus on the stability analysis of the hierarchical switched controller presented in this paper. Finally, the design of a control algorithm that considers the dynamic model of the mechanical structure could be studied when complex trajectories [38,68] must be tracked by the mobile robot.

## Figures and Tables

**Table 1:** Desired trajectory defined by Bézier. polynomials

0≤t≤1:	$\upsilon_{ref}=p_{1}\left(t\right)$		$\omega_{ref}=0$	
	${\bar{\upsilon }}_{ref}\left(t_{i}=0\right)=0;$ ${\bar{\upsilon }}_{ref}\left(t_{f}=1\right)=0.5;$			
1≤t≤2:	$\upsilon_{ref}=0.5;\;\omega_{ref}=0;$			
2≤t≤3:	$\upsilon_{ref}=p_{1}\left(t\right)$		$\omega_{ref}=p_{2}\left(t\right)$	
	${\bar{\upsilon }}_{ref}\left(t_{i}=2\right)=0.5;$ ${\bar{\upsilon }}_{ref}\left(t_{f}=3\right)=0.3;$		${\bar{\omega }}_{ref}\left(t_{i}=2\right)=0;$ ${\bar{\omega }}_{ref}\left(t_{f}=3\right)=0.9;$	
3≤t≤6.5:	$\upsilon_{ref}=0.3;\;\omega_{ref}=0.9;$			
6.5≤t≤9:	$\upsilon_{ref}=0.3;$	$\omega_{ref}=p_{2}\left(t\right)$		
		${\bar{\omega }}_{ref}\left(t_{i}=6.5\right)=0.9;$ ${\bar{\omega }}_{ref}\left(t_{f}=9\right)=-0.9;$		
9≤t≤12.5:	$\upsilon_{ref}=0.3;\;\omega_{ref}=-0.9;$			
12.5≤t≤15.8:	$\upsilon_{ref}=0.3;$	$\omega_{ref}=p_{2}\left(t\right)$		
		${\bar{\omega }}_{ref}\left(t_{i}=12.5\right)=-0.9;$ ${\bar{\omega }}_{ref}\left(t_{f}=15.8\right)=0;$		
15.8≤t≤16.8:	$\upsilon_{ref}=p_{1}\left(t\right)$		$\omega_{ref}=0;$	
	${\bar{\upsilon }}_{ref}\left(t_{i}=15.8\right)=0.3;$ ${\bar{\upsilon }}_{ref}\left(t_{f}=16.8\right)=0;$			

**Table 2:** Abrupt changes in $R_{r}$ and $R_{l}$.

$R_{m_{r}}$			$R_{m_{l}}$	
$R_{r}$	t<3s		$R_{l}$	t<3s
$9\%\;R_{r}$	3s≤t<8s		$9\%\;R_{l}$	3s≤t<8s
$R_{r}$	8s≤t<13s		$R_{l}$	8s≤t<13s
$9\%\;R_{r}$	13s≤t		$9\%\;R_{l}$	13s≤t

**Table 3:** Abrupt changes in $E_{r}$ and $E_{l}$.

$E_{m_{r}}$			$E_{m_{l}}$	
$E_{r}$	t<3s		$E_{l}$	t<3s
$65\%\;E_{r}$	3s≤t<8s		$65\%\;E_{l}$	3s≤t<8s
$E_{r}$	8s≤t<13s		$E_{l}$	8s≤t<13s
$65\%\;E_{r}$	13s≤t		$65\%\;E_{l}$	13s≤t
